# Fucoxanthin: A Promising Medicinal and Nutritional Ingredient

**DOI:** 10.1155/2015/723515

**Published:** 2015-05-27

**Authors:** Hui Zhang, Yibo Tang, Ying Zhang, Shuofeng Zhang, Jing Qu, Xu Wang, Ran Kong, Chunchao Han, Zhenquan Liu

**Affiliations:** ^1^School of Pharmacy, Shandong University of Traditional Chinese Medicine, Jinan 250355, China; ^2^School of Basic Medical Sciences, Beijing University of Chinese Medicine, Beijing 100029, China; ^3^Department of Dermatology, The First Hospital of Chinese People's Liberation Army, Gansu 730030, China; ^4^School of Chinese Materia Medica, Beijing University of Chinese Medicine, Beijing 100029, China

## Abstract

Fucoxanthin, an allenic carotenoid, can be isolated from edible brown seaweeds. Recent studies have reported that fucoxanthin has many physiological functions and biological properties, such as antiobesity, antitumor, antidiabetes, antioxidant, anti-inflammatory, and hepatoprotective activities, as well as cardiovascular and cerebrovascular protective effects. Therefore, fucoxanthin can be used as both medicinal and nutritional ingredient to prevent and treat chronic diseases. Although fucoxanthin possesses many medicinal ingredient and nutritional qualities, studies indicated that its structure was unstable. In this paper, we consulted the current documents and reviewed structural properties and factors affecting the stability of fucoxanthin. We also reported the metabolism, safety, pharmacological activities, and the methods of improving the bioavailability of fucoxanthin. Based on these studies providing essential background knowledge, fucoxanthin can be developed into marine drugs and nutritional products.

## 1. Introduction

Recently, the intake of fats, sugars, and calories is increasing, whereas exercise and physical activities are reduced. This lifestyle contributes to related diseases, like obesity, diabetes mellitus, cancer, and other chronic diseases. To prevent and treat lifestyle-related diseases, it is not sufficient to use an exclusively pharmacological treatment. Nutrition also plays critical roles [1]. Therefore, researchers begin to find safe and effective functional ingredients in food to prevent and treat lifestyle-related diseases [2]. One of these functional ingredients is fucoxanthin.

**Figure 1:**
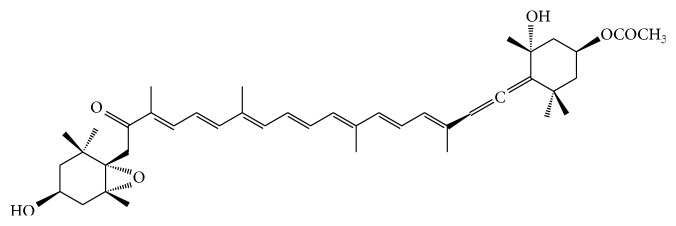
The chemical structure of fucoxanthin.

Fucoxanthin is a marine carotenoid and presents in the macroalgae and microalgae, such as* Undaria pinnatifida* (Wakame),* Laminaria japonica* (Ma-Kombu),* Phaeodactylum tricornutum*, and* Cylindrotheca closterium* [3]. The structure of fucoxanthin was determined by Englert et al. [4]. Fucoxanthin has a unique molecular structure (Figure 1) including an unusual allenic bond, a 5,6-monoepoxide, and 9 conjugated double bounds [5, 6]. The allenic bond was mainly in fucoxanthin, which was not found in other carotenoids in brown seaweeds [7]. However, the unique structure and chirality of fucoxanthin are unstable. It is easily affected by heating, aerial exposure, and illumination [8, 9]. Though fucoxanthin is unstable, the factors contributing to its instability has been thoroughly studied. Because of its unstable structure and the allenic bond, fucoxanthin showed high antioxidant activity [10]. Moreover, fucoxanthin also showed antiobesity, antidiabetes, anti-inflammatory, anticancer, and hepatoprotective activities as well as cardiovascular and cerebrovascular protective effects [11–54]. In this paper, we reviewed the factors affecting the stability of fucoxanthin, the metabolism and safety of fucoxanthin, the pharmacological activities, and the pharmacological mechanism of fucoxanthin.

## 2. Structure of Fucoxanthin

The structure of fucoxanthin is closely related to pharmacological activities of fucoxanthin. Therefore, properties of its structure are necessary to be well known. Fucoxanthin is a characteristic carotenoid, which was found in brown algae. Structure of fucoxanthin (Figure 1) is similar to neoxanthin, dinoxanthin, and peridinin. Unlike other carotenoids, fucoxanthin has a unique structure, in which an unusual allenic bond, 9 conjugated double bounds, a 5,6-monoepoxide, and some oxygenic functional groups including hydroxyl, epoxy, carbonyl, and carboxyl moieties are present [55, 56].

Like other carotenoids, fucoxanthin was ready to be degraded during storage as a result of exposure to heat, light, oxygen, enzymes, unsaturated lipids, and other prooxidant molecules [55]. The formation of some* cis*-isomers by isomerization would happen which was related to treatment conditions and medium and type of carotenoids [55–57]. Purified fucoxanthin usually resulted in three main peaks consisting of the trans-form along with two isomers [58]. The ratio of* cis*-isomers of fucoxanthin was increased with the increase of extraction temperature [58]. Kawee-ai et al. also found that when the ratio of* cis*-isomers increased, the antioxidant activities of fucoxanthin decreased [58]. A spectrophotometric analysis about fucoxanthin in canola oil was analyzed by Zhao et al. [9]. The results showed that heating caused the degradation of total and all-*trans* fucoxanthin between 25 and 100°C in the absence of light and air [9]. With the increase of heating temperature, the formation of 13-*cis* and 13′-*cis* and the degradation of 9′-*cis* would also be promoted. And the process was found to follow simple first-order kinetics. Zhao et al. [9] also found the degradation of all-*trans* and 13-*cis* and 13′-*cis* fucoxanthin was synergistically promoted when exposed to both air and light [9]. These studies provided essential background knowledge on the properties of fucoxanthin. In the process of extracting, purifying, storing, and using of fucoxanthin, the heating, aerial exposure, and illumination should be avoided as much as possible.

## 3. Metabolism and Bioavailability of Fucoxanthin

**Figure 2:**
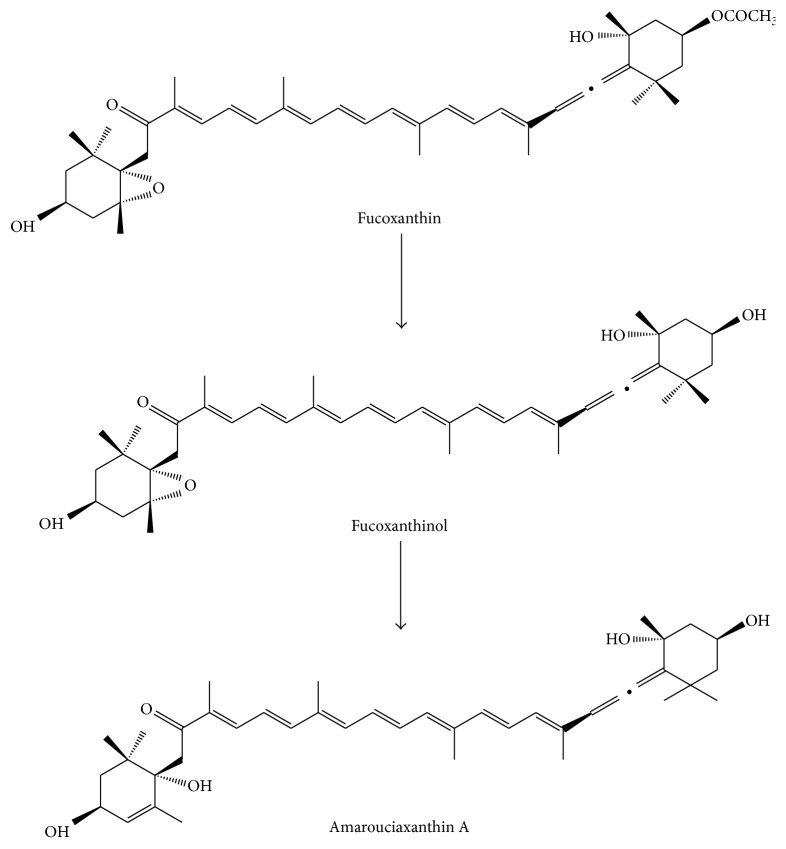
The chemical structures of fucoxanthin, fucoxanthinol, and amarouciaxanthin A.

The absorption and metabolism of fucoxanthin are closely related to its bioavailability. It is essential to know the metabolic process and the method to improve the bioavailability of fucoxanthin (Figure 2).

Fucoxanthinol and amarouciaxanthin A are the main metabolites of fucoxanthin. Fucoxanthin seemed to be rapidly hydrolyzed to fucoxanthinol in the gastrointestinal tract within 2 h after the administration and no unchanged fucoxanthin was detected in the plasma or liver in mice [59]. Fucoxanthinol was converted into amarouciaxanthin A which was predominantly shown in liver microsomes of mice and in HepG2 cells [59]. The study* in vitro* by Hashimoto et al. [60] demonstrated that dietary fucoxanthin accumulated in the heart and liver as fucoxanthinol and in adipose tissue as amarouciaxanthin A.

Yonekura et al. [61] investigated the metabolism, tissue distribution, and depletion of fucoxanthin in ICR mice. They found fucoxanthinol and amarouciaxanthin A in mice partitioned more into adipose tissues than into plasma, liver, and kidney. The half-life of the depletion (*t*_1/2_) of fucoxanthin metabolites in adipose tissues (>41 d) was longer than that in plasma (1.16 d), liver (2.63 d), and kidneys (4.44 d) [61]. In addition, they concluded that the tissue distribution of fucoxanthin metabolites was not associated with their lipophilicity, but depletion seemed to be slower because of their higher lipophilicity.

Pharmacokinetics of drugs depends on species. Mordenti [62] reported that the elimination of drugs was the fastest in mice and slowest in human subjects among the species compared. The study by Hashimoto et al. [63] showed that the bioavailability of fucoxanthinol was higher in human subjects than in mice. They also found that the metabolism of fucoxanthin differed between human subjects and mice. Fucoxanthinol is considered to be the primary active metabolite in human. And no amarouciaxanthin A was detected in the volunteer's plasma.

Solubility of fucoxanthin as an important factor must be considered for oral administration. Maeda et al. [64, 65] found fucoxanthin was difficult to dissolve in soybean oil and vegetable oils, whereas it could easily dissolve in fish oil and medium-chain triacylglycerols (MCT). The gain of white adipose tissue (WAT) weight was less in KK-*A* ^*y*^ mice fed fucoxanthin and fish oil than that in mice fed fucoxanthin alone [64]. The expression of uncoupling protein 1 (UCP1) was more clear in mice fed fucoxanthin and MCT than that in mice fed purified fucoxanthin or MCT alone [65]. These data indicated that the absorption rate of fucoxanthin could be increased by fish oil and MCT. Moreover, the study by Sugawara et al. [66] showed that lysophosphatidylcholine (lysoPC) and phospholipase A2 (PLA2) were of importance in enhancing the absorption of carotenoids in the digestive tract and supporting a simple diffusion mechanism for carotenoids assimilation by the intestinal epithelium. Thus, the absorption rate of fucoxanthin could be significantly affected by some components, especially lipids.

## 4. Safety of Fucoxanthin

Fucoxanthin is a safe pharmaceutical ingredient. Clinical research showed that taking fucoxanthin was thought to speed up metabolism, but the metabolic boost did not stimulate the central nervous system [67]. A 4-week toxicity study on repeated oral dosing of fucoxanthin (95% purity) to rats was carried out by Kadekaru et al. [68]. The results indicated that fucoxanthin did not show obvious toxicity in the rats [68]. The toxicity of the extracts containing 0.0012% fucoxanthin was determined in mice by Zaragozá et al. [69]. The extracts did not show any relevant toxicity effects in an acute toxicity test after a 4-week daily treatment. Furthermore, fucoxanthinol, the metabolite of fucoxanthin, showed no significant adverse effects* in vivo* [70].

## 5. Pharmacological Activities of Fucoxanthin

### 5.1. Antiobesity Effect

**Figure 3:**
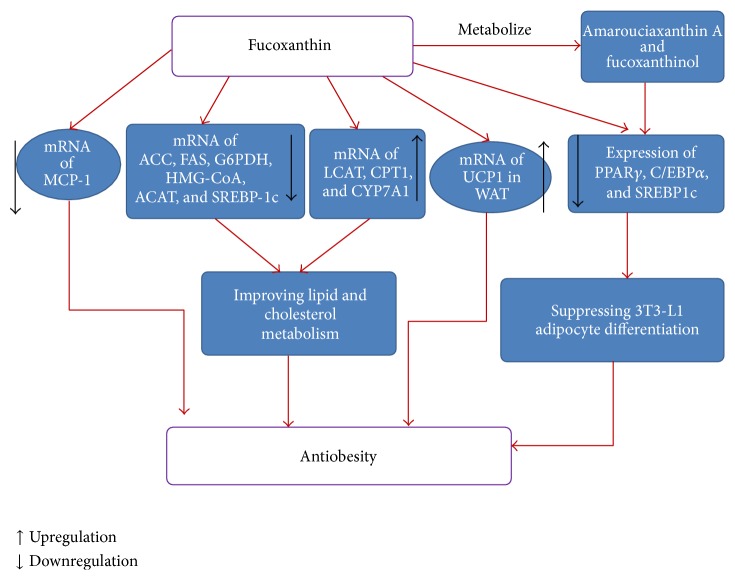
Antiobesity of fucoxanthin. ↑ Upregulation; ↓ downregulation.

Long-term consumption of high fat diets could alter lipid metabolism which lead to the accumulation of visceral fat and result in obesity and related disorders, such as diabetes mellitus, hypertension, dyslipidemia, and cardiovascular disease disorders [71, 72]. Consequently, finding efficient strategies to prevent obesity is crucial. Researchers found that fucoxanthin supplementation could play a beneficial role in antiobesity through various pathways (Figure 3).

Fucoxanthin significantly reduced plasma and hepatic triglyceride concentrations, fecal triglyceride, cholesterol, and cholesterol-regulating enzyme activities such as 3-hydroxy-3-methylglutaryl coenzyme A reductase and acyl coenzyme A [11–15].

Fucoxanthin might affect the gene expression associated with lipid metabolism to lower the level of potential lipid. Ha and Kim [16] found that fucoxanthin supplementation could decrease the mRNA expressions of hepatic acetyl-CoA carboxylase (ACC), fatty acid synthase (FAS), glucose-6-phosphate dehydrogenase (G6PDH), hydroxy-3-methylglutaryl coenzyme A (HMG-CoA), acyl-CoA cholesterol acyltransferase (ACAT), and SREBP-1C in rats. And the mRNA expressions of lecithin-cholesterol acyltransferase (LCAT), CPT1, and CYP7A1 were significantly high in the HF + Fxn group. Maeda et al. [17] reported that dietary administration of high fat (HF) diet resulted in expression of monocyte chemoattractant protein-1 (MCP-1) mRNA in mice. But the increased expression of MCP-1 mRNA was normalized by the fucoxanthin-rich Wakame lipids (WLs). The results suggested that WLs diet could ameliorate high fat (HF) diet induced lipid metabolism disorders in mice. Woo et al. [11] discovered that the activities of two key cholesterol regulating enzymes, acyl coenzyme A: cholesterol acyltransferase and 3-hydroxy-3-methylglutaryl coenzyme A reductase, were significantly inhibited by fucoxanthin in mice. Relative mRNA expressions of acyl-coA oxidase 1, palmitoyl (ACOX1), and peroxisome proliferator-activated receptor *α* (PPAR*α*) and *γ* (PPAR*γ*) were also obviously altered by fucoxanthin in the liver.

Recent studies showed that progression of 3T3-L1 preadipocyte differentiation is divided into early (days 0–2, D0–D2), intermediate (days 2–4, D2–D4), and late stages (day 4 onwards, D4-) [18]. Fucoxanthin presents different effects on 3T3-L1 cells during the three differentiation stages. Kang et al. [19] reported that when fucoxanthin presented during the early stage of differentiation (D0–D2), it promoted 3T3-L1 adipocyte differentiation and increased protein expressions of peroxisome proliferator-activated receptor *γ* (PPAR*γ*), CCAAT/enhancer-binding protein *α* (C/EBP*α*), sterol regulator element-binding protein 1c (SREBP1c), aP2, and adiponectin. However, fucoxanthin showed the inhibition to intercellular lipid accumulation by reducing the expressions of PPAR*γ*, C/EBP*α*, and SREBP1c during the intermediate (D2–D4) and late stages (D4–D7) of differentiation [19]. In addition, fucoxanthinol, the metabolite of fucoxanthin, downregulated PPAR*γ* and exhibited stronger suppressive effects than fucoxanthin on adipocyte differentiation in 3T3-L1 cells [20]. Amarouciaxanthin A, another metabolite of fucoxanthin, also showed the suppression to the expressions of PPAR*γ* and C/EBP*α* during adipocyte differentiation. Furthermore, amarouciaxanthin A showed stronger suppressive effect on glycerol-3-phosphate dehydrogenase (GPDH) activity than fucoxanthinol. Compared with fucoxanthinol, amarouciaxanthin A markedly downregulated the mRNA expressions of adipocyte fatty acid binding protein (aP2), lipoprotein lipase (LPL), and glucose-transporter 4 (Glut4) in 3T3-L1 cells [21].

Many studies suggested that fucoxanthin played an antiobesity effect by stimulating the expression of uncoupling protein 1 (UCP1) in white adipose tissue (WAT). UCP1 is usually found in brown adipose tissue (BAT) which is not expressed in WAT without stimulation. However, Maeda et al. [22] detected clear signals of UCP1 protein and mRNA in WAT when the mice were fed* Undaria pinnatifida* lipids containing fucoxanthin. Furthermore, they also [64] discovered that 0.2% fucoxanthin in the diet significantly attenuated the gain of WAT weight in KK-*A* ^*y*^ mice with increasing UCP-1 expression.

Fucoxanthin could stimulate the *β*-oxidation activity and inhibit the phosphatidate phosphohydrolase activity resulting in a decrease in the hepatic lipid droplet accumulation [12]. High fat diet induced the decrease in phosphorylation of AMP-activated protein kinase (AMPK) and acetyl-CoA carboxylase (ACC). The decrease could be restored by fucoxanthin with increasing LKB1 phosphorylation in mature 3T3-L1 adipocytes [23].

Fucoxanthin might alter plasma leptin level. Leptin secretions are elevated by the accumulation of fat in adipocytes. Leptin could control body weight and adipose fat pad through the regulation of the energy expenditure [24]. Park et al. [12] evaluated the beneficial effect of* Undaria pinnatifida* ethanol extract (UEFx) in C57BL/6J mice. They found that fucoxanthin could significantly decrease plasma leptin level which was associated with a significant decrement of the epididymal adipose tissue weight.

One study conducted in human suggested the effects of fucoxanthin on weight loss. The combination of 300 mg pomegranate seed oil and 300 mg brown seaweed extract containing 2.4 mg fucoxanthin significantly resulted in the reduction of body weight and liver fat content in obese women who were treated for 16 days [25].

### 5.2. Antitumorigenic Activity

Chemotherapy is a conventional way to decrease the rate of cancer mortality. However, the recurrence and morbidity of cancer could not be decreased through chemotherapy. Therefore, it is essential to find a promising approach to control the development of cancer. Fucoxanthin can be an effective way to control malignancies by inducing cell cycle arrest and apoptosis.

#### 5.2.1. Cell Cycle Arrest

GADD45 is involved in growth suppression. PCNA is a normal component of cyclin-dependent kinases (Cdk) complexes and a protein involved in DNA replication and repair. Smith et al. [26] previously found that when GADD45 bound to PCNA, it would stimulate DNA excision repair* in vitro* and inhibit entry of cells into S phase. In addition, GADD45A enhanced the interaction between *β*-catenin and Caveolin-1, which induced *β*-catenin translocation to cell membrane resulting in cell-cell adhesion/contact inhibition [27]. Yoshiko and Hoyoko [28] found that fucoxanthin markedly induced GADD45A in HepG2 and DU145 cells at the G1 arrest. The induction of GADD45A expression and G1 arrest by fucoxanthin was positively regulated by inhibiting p38 MAPK pathway in HepG2 cells and negatively regulated by inhibiting SAPK/JNK pathway in DU145 cells [29]. In addition, inhibition of ERK by fucoxanthin only enhanced GADD45A expression and had no influences on G1 arrest in HepG2 cells. These results suggested that different patterns of MAPK involvement in the induction of GADD45A and G1 arrest by fucoxanthin were associated with the cell type.

**Figure 4:**
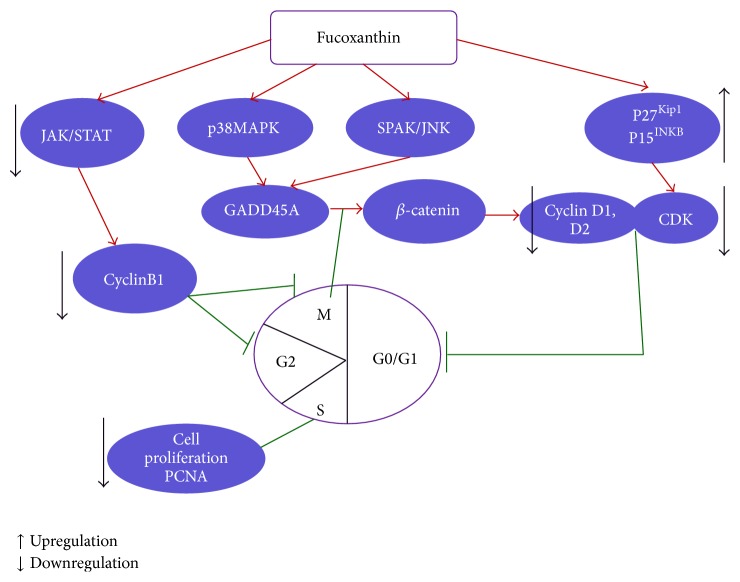
Fucoxanthin on cell cycle arrest. ↑ Upregulation; ↓ downregulation.

The study by Kim et al. [30] demonstrated that fucoxanthin decreased the proliferation of B16F10 cells accompanied by the induction of cell cycle arrest during the G(0)/G(1) phase. The cell cycle arrest during the G(0)/G(1) phase induced by fucoxanthin was related to a significant decrease in the protein expressions of phosphorylated-Rb (retinoblastoma protein), cyclin D (1 and 2), and cyclin-dependent kinase (CDK4) and significant upregulation of the protein levels of p15^INK4B^ and p27^Kip1^ [30]. Yu et al. [31] reported that fucoxanthin induced apoptosis in human gastric adenocarcinoma MGC-803 cells and cell cycle arrest in G2/M phase. Fucoxanthin markedly decreased the expressions of CyclinB1, surviving, and STAT3 in MGC-803 cells in a dose-dependent manner [31]. They also found that fucoxanthin could suppress the expression of CyclinB1 through the JAK/STAT signal pathway [31]. The mechanism of cell cycle arrest induced by fucoxanthin was described in a picture (Figure 4).

#### 5.2.2. Apoptosis Induced by Fucoxanthin and Fucoxanthinol

Apoptosis is a process of physiological cell removal which regulates the balance between cell proliferation and cell death [32]. The induction of apoptosis is now considered to be an effective way for cancer therapy [33, 34].

Recent studies revealed that STATs played an important role in maintaining EGFR-mediated cancer cell proliferation [35, 36]. Thus, inhibiting EGFR in conjunction with STATs would be a promising and attractive therapeutic strategy for cancers [37]. Wang et al. [38] investigated the antitumor mechanisms of fucoxanthin on xenografted sarcoma 180 (S180) in mice. The results showed that fucoxanthin inhibited the expressions of bcl-2, EGFR, STAT3, and phosphorylated STAT3 proteins and enhanced the expression of cleaved caspase-3. Therefore, through downregulating STAT3/EGFR signaling, fucoxanthin can induce apoptosis in S180 xenografts-bearing mice.

Both fucoxanthin and its metabolite, fucoxanthinol, can induce apoptosis. Ishikawa et al. [70] found that fucoxanthin and fucoxanthinol could inhibit cell viability of HTLV-1-infected T-cell lines and ATL cells and induce apoptosis by reducing the expressions of Bcl-2, XIAP, cIAP2, and survivin. Furthermore, uninfected cell lines and normal peripheral blood mononuclear cells were resistant to fucoxanthin and fucoxanthinol. The study by Yamamoto et al. [39] showed that fucoxanthin and fucoxanthinol induced cell cycle arrest during G1 phase and caspase-dependent apoptosis in primary effusion lymphoma cells. The apoptosis-inducing activity of fucoxanthinol was more potent than that of fucoxanthin.

### 5.3. Antidiabetic Activity

In general, consuming nutritionally rich diets and irrational dietary habits could result in obesity and diabetes mellitus. Diabetes mellitus is usually caused by obesity because excessive energy intake and accumulation of lipids can elevate insulin resistance [40]. Fucoxanthin was demonstrated to play an important role in reducing insulin resistance and blood glucose.

Saturated fat intake can elevate the HbA1c level [41]. The HbA1c level is a risk indicator of glycemia and diabetic complications [42]. The study of Woo et al. [11] suggested that supplementation of 0.05% and 0.2% fucoxanthin significantly reduced the blood HbA1c and plasma insulin level compared with the control group.

The adipokine tumor necrosis factor-*α* (TNF*α*) is involved in the development of type 2 diabetes. TNF*α* is elevated in obesity and positively associated with insulin resistance [43, 44]. The study by Maeda et al. [64] showed that 0.2% fucoxanthin improved insulin resistance and markedly decreased the blood glucose and plasma insulin concentrations in KK-*A* ^*y*^ mice by downregulating TNF*α* mRNA. Maeda et al. [17] also investigated that the fucoxanthin-rich Wakame lipids (WLs) diet could ameliorate insulin resistance by promoting expression of glucose transporter 4 (GLUT4) mRNA in skeletal muscle tissues.

Blood glucose level was positively associated with hepatic gluconeogenic enzyme activities but negatively associated with hepatic glucokinase activity [12]. Park et al. [12] found that fucoxanthin could decrease insulin resistance by elevating the ratio of hepatic glucokinase/glucose-6-phosphatase and glycogen content.

### 5.4. Antioxidative Activity

Fucoxanthin is considered as a potential antioxidant because of its unique chemical structure including an allenic bond, epoxide group, and hydroxyl group [10]. Previous studies showed that fucoxanthin possessed an effective radical scavenging ability [45, 58]. Kawee-ai et al. [58] discovered that fucoxanthin showed strong activity against BchE, with an IC50 value of 1.97 Mm and mixed inhibition type. But fucoxanthin exhibited weak activity against AChE. When the percent of* cis*-isomer increased by 2%, the scavenging activity against DPPH, superoxide anion, hydrogen peroxide, and reducing power decreased by 21.0, 10.3, 16.0, and 19.7%, respectively [58]. The antioxidant effect of fucoxanthin was determined by Ha et al.* in vivo* [46]. In experiments, the markers of antioxidant capacity like plasma total antioxidant capacity (TAC) and activities of antioxidant enzymes, such as catalase, superoxide dismutase (SOD), were determined in rats fed HF diet and HF + Fxn diet. Glutathione peroxidase (GSH-Px) and mRNA expressions of transcription factor and nuclear erythroid factor like 2 (Nrf2), as well as its target genes such as NAD(P)H quinone oxidoreductase 1 (NQO1), were also determined. The result showed that the activity of GSH-Px in plasma and liver was both significantly elevated by fucoxanthin supplementation. Plasma TAC level and mRNA expressions of Nrf2 and NQO1 were also obviously higher in the HF + Fxn group than those in the HF group [46].

### 5.5. Anti-Inflammatory Activity

Previous studies determined that anti-inflammatory agents should reduce the inflammatory response by suppressing the inflammatory mediators including nutric oxide (NO), prostaglandin E_2_ (PGE_2_), tumor necrosis factor-*α* (TNF*α*), interleukin- (IL-) 1*β*, IL-6, and inflammatory cytokines such as cyclooxygenase (COX) and inducible nutric oxide synthase (iNOS) [47]. Kim et al. [48] investigated the anti-inflammatory effect of fucoxanthin in lipopolysaccharide- (LPS-) stimulated murine macrophage RAW 264.7 cells. The results demonstrated that fucoxanthin could reduce the levels of proinflammatory mediators including NO, PGE_2_, IL-1*β*, TNF*α*, and IL-6 by suppressing the NF-*κ*B activation and the MAPK phosphorylation. Additionally, fucoxanthin reduced the levels of iNOS and COX-2 proteins in a dose-dependent manner [48]. Sakai et al. [49] authenticated the anti-inflammatory and antiallergenic properties of fucoxanthin* in vivo*. The result showed that fucoxanthin could markedly inhibit the antigen-induced release of *β*-hexosaminidase in rat basophilic leukemia 2H3 cells and bone marrow-derived mast cells. Furthermore, fucoxanthin suppressed antigen-induced aggregation of the high affinity IgE receptor (Fc ∈ RI) and Fc ∈ RI-mediated intracellular signaling. It suggested that fucoxanthin inhibited the degranulation of mast cells by suppressing the aggregation of Fc ∈ RI [49].

### 5.6. Hepatoprotective Effect

Increasing fatty acid oxidation and decreasing the amounts of fatty acids as substrates for triacylglycerol synthesis could improve fatty liver [12]. Fucoxanthin could increase fatty acid oxidation and decrease the hepatic lipid contents by regulating activities of hepatic lipid metabolic enzymes and stimulating *β*-oxidation activity [11, 12]. Woo et al. [11] found that fucoxanthin supplementation significantly decreased the hepatic lipid contents and the concentration of plasma triglyceride in C57BL/6N mice. The mechanism is that fucoxanthin inhibited the activities of hepatic lipogenic enzymes, glucose-6-phosphate dehydrogenase, malic enzyme, fatty acid synthase, and phosphatidate phosphohydrolase as well as elevated *β*-oxidation activity. Furthermore, Park et al. [12] also demonstrated that fucoxanthin supplementation stimulated *β*-oxidation activity and reduced the phosphatidate phosphohydrolase activity resulting in the hepatic lipid droplet.

Docosahexaenoic acid (DHA) is an important n-3 functional polyunsaturated fatty acid in biological systems [50]. Fucoxanthin was reported to promote the proportion of DHA and AA in liver lipids of mice [50–52]. DHA and AA contents in the liver lipids could be also enhanced by fucoxanthinol [51, 52].

Liu et al. [53] demonstrated that pretreatment with fucoxanthin (1–20 *μ*M) for 24 h could protect the murine hepatic BNL CL.2 cells against oxidative damage induced by ferric nitrilotriacetate (Fe-NTA). They [53] found that when the incubation of BNL CL.2 cells was treated with Fe-NTA for 30 min, cell proliferation was obviously decreased. However, fucoxanthin significantly recovered cell proliferation in a dose-dependent manner. Furthermore, the data suggested that fucoxanthin not only decreased the level of thiobarbituric acid-reactive substances (TBARS) and protein carbonyl contents but also increased the level of GSH in a concentration-dependent manner. All the results indicated that fucoxanthin could inhibit cytotoxicity in hepatic BNL CL.2 cells induced by Fe-NTA.

### 5.7. Cardiovascular and Cerebrovascular Protective Effects

A lipid profile is a risk factor of cardiovascular disease. Fucoxanthin can improve the lipid profile and prevent the damage in cardiovascular system by promoting the proportion of DHA in the liver [12].

Hypertension is associated with cerebrovascular diseases. Ikeda et al. [54] examined the effect of Wakame on the development of stroke in stroke-prone spontaneously hypertensive rats (SHRSP). They found that the development of stroke signs was significantly delayed and the survival rate of SHRSP was significantly improved by Wakame. However, the blood pressure showed no significant difference among groups. The results indicated that Wakame improved cerebrovascular diseases in SHRSP, independent of hypertension. They also found that fucoxanthin isolated from Wakame may have a preventive effect on ischaemic cultured neuronal cell death. In addition, fucoxanthin could obviously attenuate neuronal cell injury in hypoxia and reoxygenation.

## 6. Conclusion

Fucoxanthin is a special carotenoid and has many bioactivities. The results of animal studies showed that fucoxanthin had potential value in preventing and treating lifestyle-related diseases, as obesity, diabetes, cancer, cardiovascular disease, and other chronic diseases. Though there are a few studies in human subjects, more clinical trials should be conducted. Fucoxanthin is proved to have no side effects and can be easily extracted from macroalgae and microalgae. Additionally, the methods to improve its stability and bioavailability have been studied. With previous documents and further clinical researches, fucoxanthin will be easier to be developed into safe marine drugs and nutritional products to prevent and treat lifestyle-related diseases.
